# A French translation and validation of the Durand Adaptive Psychopathic Traits Questionnaire: An investigation with community samples from France and Canada

**DOI:** 10.1371/journal.pone.0204214

**Published:** 2018-09-26

**Authors:** Guillaume Durand

**Affiliations:** Department of Psychiatry and Neuropsychology, Faculty of Health, Medicine, and Life Sciences, Maastricht University, Maastricht, The Netherlands; University of La Rioja, SPAIN

## Abstract

This study presents a French translation and validation of the Durand Adaptive Psychopathic Traits Questionnaire (DAPTQ; Durand, 2017), an instrument for assessing adaptive traits known to correlate with the psychopathic personality. The first sample, which included individuals from France and Canada (*N* = 135, 52% in France, *M*_age_ = 26.98, *SD* = 9.24), completed the DAPTQ in French, alongside measures of empathy, positive and negative affects, satisfaction with life, and self-esteem. The second sample included bilingual (French and English) individuals from France and Canada (*N* = 141, 52% in France, *M*_age_ = 29.73, *SD* = 9.09) who completed both versions of the DAPTQ (French and English), alongside measurements of perceived stress, trait anxiety, and creativity. The results support the DAPTQ–French version good internal consistency (α = .89/.87), convergent validity, and concurrent validity. Correlation between the DAPTQ total and subscales across versions (French-English) showed strong associations (*r* = .84 to .96). These findings support the cross-cultural equivalence of the DAPTQ and its effectiveness as a valid assessment method of adaptive traits in the field of psychopathy.

## Introduction

Among the many controversies in the field of psychopathy, the existence of a subtype encompassing adaptive traits is highly debated [1–4]. Upon providing a definition of psychopathy, many researchers emphasize the negative traits associated to psychopathy, such as impulsivity, dishonesty, lack of empathy, and aggression [3,5–8]. These definitions, characterizing psychopaths as violent, ruthless, criminally prone and irrecoverable individuals, diverge from the initial conception of psychopathy proposed by Cleckley [9], acknowledging successful psychopaths. Successful psychopaths were initially defined as individuals possessing core psychopathic traits (such as lack of empathy and emotional detachment), but also possessing adaptive traits, such as social charm, low neuroticism, and stress/anxiety resilience [10–12]. Although the inclusion of the aforementioned adaptive components as key structures of psychopathy is highly debatable, numerous studies provided evidence of a relationship between an increase of psychopathic traits and adaptive traits [13–22].

While most instruments assessing psychopathy and psychopathic traits exclusively focus on maladaptive traits, two well-validated measures of psychopathic traits partially examine adaptive traits. The Psychopathic Personality Inventory (PPI) divides eight psychopathic traits into two main factors, namely PPI-I (fearless dominance) and PPI-II (impulsive antisociality) [23]. More specifically, PPI-I focuses on three adaptive characteristics, namely social charm, stress and anxiety immunity, and fearlessness. Although the relationship between PPI-I and psychopathy is highly debated [4,6,24,25], results nevertheless suggest that high levels of PPI-I are associated with numerous adaptive traits, such as lower provoked violence [17], higher levels of self-esteem and stable happiness [22,26], and emotional stability [20]. Similarly to the PPI, the Triarchic Psychopathy Measure (TriPM) includes a component, namely boldness, measuring the adaptive side of psychopathy [11]. Previous studies support a strong correlation between PPI-I and boldness (*r* = .82) [27].

While the PPI and the TriPM are commonly used to examine psychopathy from a maladaptive and adaptive point of view, past findings do not completely support the efficiency of either instruments to measure successful psychopathy. Multiple researchers have identified distinctive personality traits in successful psychopathy, such as high levels of extroversion, high conscientiousness, low agreeableness, high leadership abilities, high communication abilities, a willingness to take risks, and an immunity to stress and anxiety [28–30]. Although the PPI and the TriPM are associated with numerous of the aforementioned characteristics, neither are associated with conscientiousness [31,32]. Furthermore, the spectrum of adaptive traits measured by the PPI and the TriPM is limited. Several characteristics initially proposed by Cleckley as common traits observed in psychopaths are not assessed by the PPI or the TriPM (i.e. absence of: delusions, irrational thinking, depression, mood swings, and worries) [33]. Altogether, these findings suggest that the PPI and the TriPM could benefit from a complementary questionnaire focusing solely on extending PPI-I and boldness.

The Durand Adaptive Psychopathic Traits Questionnaire (DAPTQ; Durand, 2017) was developed in order to increase the predictive power of either instruments when exploring successful psychopathy [34]. The DAPTQ is a self-report instrument assessing eight adaptive personality traits that have shown previous associations with the psychopathic personality. The DAPTQ was developed by first identifying all constructs considered adaptive, defined as ‘‘a trait maximizing an individual’s survival probability within a set environment”, which have been associated with the concept of psychopathy in healthy adults. A pool of 19 distinct constructs emerged. After an examination of internal consistency and exploratory factor analysis, confirmed with a parallel analysis, an 11-factor solution emerged. Additional validation studies excluded two factors due to a lack of association with the total the total score. Subsequent research failed to provide support for one of its subscale, namely ‘Money Smart’, leaving a final solution of 38 items divided within eight factors (leadership, logical thinking, composure, creativity, fearlessness, focus, extraversion, and management) [35]. The DAPTQ is not used to diagnose psychopathy, nor does it identify highly psychopathic individuals. Instead, the DAPTQ focuses on a wide range of adaptive traits theorized to be associated, either centrally or peripherally, to the concept of successful psychopathy, and should be seen as an extension of PPI-I and Boldness [35].

A validation study of the DAPTQ provided support for the usage of the DAPTQ in conjunction with the PPI or the TriPM [35]. In the first study, the DAPTQ provided significant incremental validity over the PPI-SF when measuring core personality traits from the Big Five. Furthermore, while PPI-I was not associated with conscientiousness, the DAPTQ total score was weakly correlated with the construct (*r* = .24). In the second study, the DAPTQ demonstrated incremental validity over both the PPI-SF and the TriPM on measures of communication adaptability, perceived stress, and trait anxiety.

Previous studies focusing on the DAPTQ have, up to now, exclusively focused on worldwide sample pools, combining participants from all nationalities in single samples. Multiple studies demonstrate that the expression of psychopathy significantly differs across countries and cultures [36,37]. Furthermore, all studies related to the DAPTQ have been conducted in English. Examining the validity and reliability of a translated version of the DAPTQ in a heterogeneous population would provide additional information regarding the validity of the DAPTQ as a culture-independent instrument.

The present study hence aims to investigate the psychometric properties of the French translation of the DAPTQ in francophones, specifically those located in Canada or France. Similarly to American and British English, Canadian and France French possess a few linguistic differences. Validating the French version of the DAPTQ in both of these samples will provide additional data regarding the effect of culture on the DAPTQ. Therefore, two studies will be performed. The first study will focus on the cultural difference between Canadians and French on the DAPTQ, as well as its interaction with traits considered adaptive. The second study will focus on bilingual French-English Canadians and French, and will examine the differences between the French and the English version of the DAPTQ. The second study will also examine potential differences between various adaptive traits used in the current study and also used in a previous validation of the DAPTQ [35].

## Method of Study 1

### Participants

The following studies were approved and given ‘exempt’ status by the IntegReview Ethical Review Board (Austin, TX, USA), under protocol number 11022016. Before beginning the study, all participants provided informed consent online by selecting the option ‘I consent to participate in this study’ following the explanation of the research and their rights as participants. A total of 142 participants were recruited from social media and websites dedicated to psychological research. Inclusion criteria were to be a native speaker in French, to be over 18 years old, and to be located in either France or Canada. Examination of potential outliers was done by analyzing the stem-and-leafs plot for each subscale used in the present study. Those analyses identified 7 potential outliers, which were removed from the study. The remaining 135 participants consisted of 71 males and 64 females. Participants location was evenly distributed, with 48% of participants located in Canada, and 52% located in France. Participants’ gender was also well distributed between location, with 47% of males being in Canada (53% males in France), and 50% of females being in Canada (50% females in France). Most participants reported being of Caucasian ethnicity (89%). A total of 56 participants (42%) reported being currently enrolled as a student in a university. In terms of education, most participants reported having received a High school diploma (42%), a Master’s degree (24%), a bachelor’s degree (20%), or other (14%). The participants mean age was 26.98 years old (*SD* = 9.24).

### Measures

#### Durand adaptive psychopathic traits questionnaire (DAPTQ; Durand, 2017)

The DAPTQ is a 38-item self-reported questionnaire, rated from 1 = *Strongly Disagree* to 6 = *Strongly Agree*. The DAPTQ provides a total score, along 8 subscales scores: Leadership, Logical Thinking, Composure, Creativity, Fearlessness, Focus, Extroversion, and Management. A higher score represents higher adaptive traits.

#### Interpersonal reactivity index–french version (IRI; Gilet, Mella, Studer, Grühn, Labouvie-Vief, 2013)

The IRI–French Version is a translation from the original IRI by Davis [38]. The instrument is a 28-item self-reported questionnaire assessed on a 7-point scale, and measures four components of empathy; empathic concern (EC) and personal distress (PD) focus on the affective aspects of empathy, while fantasy (FS) and perspective taking (PT) focus on the cognitive aspects. The EC scale measures one’s tendency to experience feelings of concern or compassion for others. The PD scale measures one’s tendency to experience distress or discomfort in response to others’ emotional distress. The FS scale measures one’s tendency to get involved in fictional situations and to identify with fictional characters in books, movies, or play. Lastly, the PT scale measures one’s tendency to adopt another’s perspective or point of view. The French version of the IRI has shown evidence of good reliability and convergent validity with related constructs [39].

#### Positive and negative affective states–french version (PANAS; Gaudreau, Sanchez, Blondin, 2006)

The PANAS–French Version is a translation from the original PANAS by Watson, Clark, and Tellegen [40]. The PANAS is a self-reported adjective checklist containing two 10-item subscales measuring positive (i.e., active, alert, attentive, determined, enthusiastic, excited, inspired, interested, proud, and strong) and negative (i.e., afraid, ashamed, distressed, guilty, hostile, irritated, jittery, nervous, scared, and upset) affects. The French version of the PANAS has shown evidence of good reliability and convergent validity with related constructs [41].

#### Satisfaction with life scale–french version (SWLS; Blais, Vallerand, Pelletier, Brière, 1989)

The SWLS–French Version is a translation from the original SWLS by Diener, Emmons, Larsen, and Griffin [42]. The SWLS measures life satisfaction using 5 items rated on a 7-point Likert scale. The French version of the SWLS has shown evidence of adequate psychometric norms [43].

#### Sate self-esteem scale–french version (SSES; Martinot, Redersdorff, 2003)

The SSES–French Version is a translation from the original SSES by Heatherton and Polivy [44]. The SSES measures an individual’s current self-esteem, in the present moment. The SSES is scored using a total score, as well as three subscale scores: performance (i.e., I feel confident about my abilities), social (i.e., I feel self-conscious), and appearance (i.e., I feel that others respect and admire me). The French version of the SSES has shown evidence of acceptable reliability [45].

### French translations

The DAPTQ was translated to French by the original author of the DAPTQ, and was back-translated by two researchers, one being familiar with the DAPTQ, the other one being unfamiliar with it. The back-translations were reviewed by the original author, confirming the previous translation to French. No further change was done to the initial translation.

## Results

### Preliminary analyses

With the exception of the SSES, all participants fully completed all questionnaires, and hence no missing data was reported. A programming mistake resulted in only 98 participants completing the SSES. There was no missing data on the SSES within those 98 participants. A normal distribution was reported on all scales and subscales, with a Skewness range between -0.73 and 0.60 and a Kurtosis range between -0.93 and 0.15. No scale or item was further transformed.

### Location differences

Examination of the DAPTQ total score by location did not identified any significant mean difference. Further analysis of the DAPTQ subscales, however, identified a few differences. Individuals in Canada reported higher scores on the Leadership subscale (*F*(1, 134) = 4.076, *p* = .046, *d* = 0.35), on the Creativity subscale (*F*(1, 134) = 5.024, *p* = .027, *d* = 0.39), and the Extroversion subscale (*F*(1,134) = 5.354, *p* = .022, *d* = 0.40). Examination of the SSES, PANAS, IRI, and SWLS allowed in finding one additional difference, with Canadians scoring higher on the IRI–PT scale (*F*(1, 134) = 5.115, *p* = .025, *d* = 0.39).

### Correlations between the DAPTQ and personality measures

In order to account for multiple testing and potential type I error, the criterion of *p* < .01 was used to establish statistical significance for all correlational analysis. Table 1 shows the inter-scale correlations of the DAPTQ, as well as the mean, standard deviation, and Cronbach’s alpha of each subscales. Similarly to previous studies, all subscales of the DAPTQ correlated moderately to strongly with the DAPTQ total score [34,35]. Table 2 shows the association between the IRI, the PANAS, the SWLS, and the SSES with each of the DAPTQ’s subscales. The DAPTQ total score was negatively associated with IRI–PD (*r* = -.57), positively associated with PANAS-Positive (*r* = .34) but negatively with PANAS-Negative (*r* = -.53), positively associated with SWLS (*r* = .23) and positively associated with the SSES total and all its subscales (*r* = .45 to .56).

**Table 1:** Inter-correlations between the DAPTQ subscales in study 1 (*N* = 135).

Scales	1	2	3	4	5	6	7	8	Mean (SD)	α
DAPTQ										
1. Leadership									13.84 (3.80)	.79
2. Logical Thinking	.16								21.65 (4.32)	.81
3. Composure	**.27**	**.46**							17.25 (7.34)	.91
4. Creativity	**.31**	-.05	.09						16.01 (5.10)	.88
5. Fearlessness	.17	.11	**.22**	.15					19.38 (6.11)	.82
6. Focus	.05	**.36**	**.34**	.03	.06				11.20 (3.93)	.85
7. Extroversion	**.44**	-.03	**.29**	**.28**	.17	.12			17.08 (6.44)	.81
8. Management	**.34**	**.41**	**.46**	.12	-.04	**.57**	**.27**		10.28 (3.39)	.71
9. DAPTQ Total	**.58**	**.50**	**.74**	**.44**	**.47**	**.50**	**.61**	**.62**	126.71 (22.88)	.89

Note. Bold indicates p < .01, two-tailed. 1 = Leadership; 2 = Logical Thinking; 3 = Composure; 4 = Creativity; 5 = Fearlessness; 6 = Focus; 7 = Extroversion; 8 = Management; 9 = DAPTQ Total.

**Table 2:** Correlation between the DAPTQ—French version and personality traits.

Scales	1	2	3	4	5	6	7	8	9	Mean (SD)	α
IRI (*N* = 135)											
Perspective taking	-.05	.19	.16	.20	.03	.16	.05	.09	.18	25.82 (4.75)	.82
Fantasy scale	.08	**-.37**	**-.36**	**.22**	-.13	-.18	.02	-.19	-.21	25.39 (5.25)	.80
Empathic concern	-.11	**-.26**	**-.24**	.21	-.14	.01	-.10	-.09	-.17	25.37 (5.67)	.87
Personal distress	**-.38**	**-.48**	**-.46**	-.07	**-.34**	**-.28**	**-.22**	**-.38**	**-.57**	19.14 (5.32)	.83
PANAS (*N* = 135)											
Positive	**.42**	.11	.21	**.29**	.11	.07	.14	**.25**	**.34**	29.22 (6.69)	.83
Negative	-.14	**-.41**	**-.69**	.01	-.06	**-.37**	-.21	**-.47**	**-.53**	21.80 (8.04)	.88
SWLS (*N* = 135)											
Total	**.26**	.13	**.32**	-.03	-.13	.13	.08	**.39**	**.23**	20.45 (7.61)	.88
SSES (*N* = 98)											
Performance	**.48**	**.36**	**.45**	.23	-.08	**.29**	.20	**.58**	**.53**	25.20 (6.14)	.84
Social	.21	**.35**	**.56**	.13	-.11	**.27**	.17	**.43**	**.45**	22.44 (6.90)	.88
Appearance	**.30**	**.27**	**.50**	.01	.04	.20	**.39**	**.35**	**.48**	17.30 (5.23)	.83
Total	**.38**	**.38**	**.58**	.15	-.07	**.30**	**.28**	**.53**	**.56**	64.95 (15.84)	.92

Note. Bold indicates p < .01, two-tailed. 1 = Leadership; 2 = Logical Thinking; 3 = Composure; 4 = Creativity; 5 = Fearlessness; 6 = Focus; 7 = Extroversion; 8 = Management; 9 = DAPTQ Total.

## Method of Study 2

### Participants

A total of 153 participants were recruited from social media and websites dedicated to psychological research. Inclusion criteria were to be bilingual in French and English, to be over 18 years old, and to be located in either France or Canada. Examination of potential outliers was done by analyzing the stem-and-leafs plot for each subscale used in the present study. Those analyses identified 12 potential outliers, which were removed from the study. The remaining 141 participants consisted of 95 males and 46 females. Participants location was evenly distributed, with 48% of participants located in Canada, and 52% located in France. Participants’ gender was also well distributed between location, with 44% of males being in Canada (56% males in France), and 57% of females being in Canada (43% females in France). Most participants reported being of Caucasian ethnicity (90%). The participant’s mother tongue was either French (70%) or English (30%). When examined by country, the mother tongue of Canadians was mostly equal (53% English, 47% French), while French’s mother tongue was mostly French (89% French, 11% English). A total of 50 participants (36%) reported being currently enrolled as a student in a university. In terms of education, most participants reported having received a Master’s degree (31%), a bachelor’s degree (27%), a High school diploma (19%), a Doctoral degree (9%) or other (14%). The participants mean age was 29.73 years old (*SD* = 9.09).

### Measures

The original DAPTQ–English version was used at the beginning of the study, and the DAPTQ–French version was used at the end of the study.

#### Perceived stress scale–french version (PSS; Cohen, Kamarck, & Mermelstein, 1983)

The PSS is a 10-item self-reported instrument assessing perceived stress in everyday situations [46]. The questionnaire is rated on a 5-point Likert scale (0 = *Never* to 4 = *Very Often*). The French version has been validated in previous samples and offers evidence of adequate internal consistency and validity [47,48].

#### State-trait anxiety inventory–trait version (STAI-Y2; Spielberger, Gorsuch, & Lushene, 1970)

The STAI is a self-reported questionnaire containing 40 items divided into two subscales, assessing state anxiety and trait anxiety [49]. The STAI-Y2 subscale focuses on trait anxiety (e.g., how a participant feels in everyday life). The STAI has been used in numerous studies, and is considered to have good validity with related variables and internal consistency [50].

#### Scale of creative attributes and behavior (SCAB; Kelly, 2004)

The SCAB is a 20-item questionnaire assessing facets related to creativity using a 7-point Likert scale (1 = *Strongly Disagree* to 7 = *Strongly Agree*) [51]. The scale provides a total score and five subscales: Creative Engagement, Creative Cognitive Style, Spontaneity, Tolerance, and Fantasy.

## Results

### Preliminary analyses

All participants fully completed all questionnaires, and hence no missing data was reported. A normal distribution was reported on all scales and subscales, with a Skewness range between -0.82 and 0.34 and a Kurtosis range between -0.83 and 0.60. No scale or item was further transformed.

### Location differences

Similarly to Study 1, examination of the DAPTQ total score by location did not identified any significant mean difference for both the English version and French version of the DAPTQ total score. Further analysis of the DAPTQ subscales, however, identified a few differences. Regarding the DAPTQ–English version, individuals in Canada reported high scores on the Leadership subscale (*F*(1, 140) = 10.62, *p* = .001, *d* = 0.55), the Focus subscale (*F*(1, 140) = 4.68, *p* = .032, *d* = 0.37), and the Management subscale (*F*(1,140) = 9.72, *p* = .002, *d* = 0.53). An identical trend was observed in the DAPTQ–French version, where individuals in Canada reported higher scores on the Leadership subscale (*F*(1, 140) = 10.65, *p* = .001, *d* = 0.55), the Focus subscale (*F*(1, 140) = 6.24, *p* = .014, *d* = 0.42), and the Management subscale (*F*(1,140) = 19.25, *p* < .001, *d* = 0.74).

Aside from the mean differences by location observed in the DAPTQ, a few notable differences were observed on the STAI-Y2 and the SCAB. First, individuals from France reported higher scores on the STAI-Y2 (*F*(1, 140) = 6.98, *p* = .009, *d* = 0.45). Second, participants from Canada scored higher on two subscales of the SCAB: Cognitive Styles (*F*(1, 140) = 6.86, *p* = .010, *d* = 0.44) and Spontaneity (*F*(1, 140) = 4.25, *p* = .041, *d* = 0.35).

### Correlation between the French and the English version of the DAPTQ

As shown in Table 3, both version of the questionnaire reported adequate intercorrelation, with all subscales of the English version (*r* = .25 to .75) and of the French version (*r* = .28 to .70) correlating to their respective total score.

**Table 3:** Inter-correlations between the DAPTQ subscales by version (N = 141).

Scales	1	2	3	4	5	6	7	8	Mean (SD)	α
DAPTQ—English version										
1. Leadership									14.31 (4.02)	.87
2. Logical Thinking	-.01								23.23 (4.05)	.84
3. Composure	**.35**	**.22**							19.80 (6.95)	.90
4. Creativity	.13	-.10	-.06						15.89 (4.73)	.86
5. Fearlessness	**.24**	.02	**.39**	.02					19.28 (6.33)	.88
6. Focus	**.26**	**.27**	**.47**	-.07	**.26**				12.41 (4.50)	.91
7. Extroversion	**.64**	-.14	**.28**	.20	.19	.08			19.15 (6.76)	.86
8. Management	**.43**	**.34**	**.52**	-.02	.11	**.43**	**.24**		11.72 (2.99)	.71
9. DAPTQ Total	**.68**	**.29**	**.75**	**.25**	**.58**	**.58**	**.62**	**.62**	135.80 (22.54)	.89
Scales	1	2	3	4	5	6	7	8	Mean (SD)	α
DAPTQ—French version										
1. Leadership									14.09 (3.45)	.78
2. Logical Thinking	.03								23.02 (3.73)	.79
3. Composure	**.27**	.21							19.41 (6.42)	.88
4. Creativity	.14	-.15	-.11						15.91 (4.57)	.85
5. Fearlessness	**.28**	-.02	**.40**	.04					19.60 (5.68)	.83
6. Focus	**.26**	**.28**	**.42**	-.05	.21				12.36 (3.91)	.86
7. Extroversion	**.56**	-.11	.20	**.22**	**.22**	.11			18.99 (6.44)	.83
8. Management	**.41**	**.28**	**.42**	-.04	.08	**.48**	.15		11.65 (2.76)	.59
9. DAPTQ Total	**.66**	**.28**	**.70**	**.26**	**.59**	**.58**	**.62**	**.55**	135.05 (20.07)	.87

Note. Bold indicates p < .01, two-tailed. 1 = Leadership; 2 = Logical Thinking; 3 = Composure; 4 = Creativity; 5 = Fearlessness; 6 = Focus; 7 = Extroversion; 8 = Management; 9 = DAPTQ Total.

Correlational analysis of the DAPTQ between version support the reliability of the French version, with a correlation between the two total scores of *r* = .96. The correlations between the English and the French version by subscales are as follows: Leadership (*r* = .92), Logical Thinking (*r* = .88), Composure (*r* = .92), Creativity (*r* = .93), Fearlessness (*r* = .92), Focus (*r* = .91), Extroversion (*r* = .96), and Management (*r* = .84). Examination of the correlations between versions for each of the 38 items revealed only strong correlations (*r* = .56 to .94).

#### Correlations between the DAPTQ–French version and personality measures

As shown in Table 4, the DAPTQ–French version was correlated to two measures previously used to validate the DAPTQ in its development phase, as well as in its subsequent validation, namely the PSS and the STAI-Y2, and one additional measure assessing creativity (SCAB). Similar to the results found by Durand [34,35], the PSS was strongly negatively correlated with the DAPTQ–French version total (*r* = -.51) alongside two subscales: Composure (*r* = -.56) and Management (*r* = -.50). Only two other subscales of the DAPTQ were negatively associated with the PSS, namely Logical Thinking (r = -.28) and Focus (r = -.39). Similar results were obtained on the STAI-Y2, which showed a strong negative correlation with the DAPTQ–French version total (*r* = -.70), alongside the two subscales aforementioned: Composure (*r* = -.69) and Management (*r* = -.55). All other subscales, with the exception of Logical Thinking and Creativity, displayed a weak to moderate negative correlation with the STAI-Y2 (*r* = -.28 to -.45).

**Table 4:** Correlation between the DAPTQ—French version and personality traits (N = 141).

Scales	1	2	3	4	5	6	7	8	9	Mean (SD)	α
PSS											
Total	-.14	-**.28**	**-.56**	.03	-.17	**-.39**	-.14	**-.50**	**-.51**	17.48 (4.48)	.55
STAI-Y2											
Total	**-.43**	-.21	**-.69**	-.02	**-.28**	**-.45**	**-.35**	**-.55**	**-.70**	43.60 (11.62)	.93
SCAB											
Engagement	**.24**	-.09	.05	**.69**	.10	.11	**.31**	.07	**.34**	18.09 (5.26)	.80
Cognitive styles	**.39**	.10	-.01	**.34**	.07	.03	.13	.18	**.22**	21.02 (3.74)	.74
Spontaneity	**.36**	**-.38**	.14	**.22**	**.35**	-.03	**.41**	.05	**.26**	14.82 (4.63)	.73
Tolerance	**.26**	-.01	**.28**	.19	**.51**	.18	**.33**	.19	**.45**	26.14 (3.93)	.70
Fantasy	-.09	-.11	-.16	**.34**	-.01	-.20	.02	-.14	-.07	21.85 (4.30)	.69
Total	** .33**	-.15	.05	** .61**	** .27**	.01	**.37**	.08	** .34**	98.53 (13.24)	.81

Note. Bold indicates p < .01, two-tailed. 1 = Leadership; 2 = Logical Thinking; 3 = Composure; 4 = Creativity; 5 = Fearlessness; 6 = Focus; 7 = Extroversion; 8 = Management; 9 = DAPTQ Total.

Additional examination of the DAPTQ–French version and measurements of creativity further support the validity of the DAPTQ. Several correlations were also observed between the DAPTQ–French version and the SCAB. The DAPTQ–French version displayed positive correlations between its total score and the SCAB total and its subscales, with the exception of the fantasy subscale (*r* = .22 to .45). Examination of the DAPTQ–French version subscale identified three strong correlations. The creativity subscale was positively correlated to the SCAB total (*r* = .61) and the Engagement subscale (*r* = .69). Additionally, Fearlessness displayed a positive correlation with Tolerance (*r* = .51).

## Discussion

This study aimed to examine the validity and psychometric properties of the DAPTQ–French version in two samples by, 1) comparing the results between Canadians and French, and 2) comparing the French and English version of the DAPTQ in bilingual individuals from either France or Canada. Analyses support minor differences between French and Canadian participants in both studies. Furthermore, the DAPTQ French version and the DAPTQ English version were answered in a very similar way by the participants of Study 2. Lastly, the results obtained on the PSS and the STAI-Y2 in Study 2 are very similar to those obtained in previous studies focusing on the English version of the DAPTQ [34,35]. The addition of numerous correlates across the two studies (e.g., IRI, PANAS, SWLS, SSES, SCAB) provides additional information regarding the general psychometric properties of the DAPTQ.

Examination of the effect of location on DAPTQ’s score and other measurements yielded interesting results. In Study 1, participants in Canada reported higher levels of DAPTQ-leadership, DAPTQ-creativity, DAPTQ-extroversion, and IRI-perspective taking. In Study 2, Canadians displayed higher levels of DAPTQ-leadership, DAPTQ-focus, DAPTQ-management, SCAB-creativity, and lower levels STAI-anxiety than participants in France. The effect of location on leadership is particularly robust, being supported by both studies. Previous findings identified cultural differences in leadership, whereas Polish enterprises displayed higher authentic leadership than German enterprises [52]. Similarly to leadership, previous findings also support a cross-cultural difference of STAI scores between various cultures. One study investigated STAI scores between students from America, Turkey, Mexico, and the Philippines, and concluded that Filipino students reported the highest scores, while American students reported the lowest scores [53]. Although this study was not specifically investigating France, or even Europe, the results support overall lower levels of anxiety within Americans, who are culturally similar to Canadians.

Estimation of the reliability of the scores of the DAPTQ total score in both studies, and both versions of Study 2, was acceptable, alongside its 8 subscales. Although the Management subscale showed the weakest Cronbach’s alpha in both studies, its strong correlation with the DAPTQ total score supports its construct validity. Cronbach’s alpha for all other variables (IRI, PANAS, SWLS, SSES, STAI-Y2, and the SCAB) were also acceptable. It should however be noted that the French version of the PSS in the present study displayed poor internal consistency reliability, especially compared to the English version used during the development of the DAPTQ, which reported an alpha of .89 [34].

Inter-construct validation was performed by correlating all DAPTQ subscales to its total scores. Although correlations were slightly weaker in the French version, both versions of the DAPTQ displayed a significant correlation between each of the subscales and their respective total score. Validation of the DAPTQ–French version was further supported by correlating each subscale of both versions, with no Pearson correlation under *r* = .84, and no Pearson correlation under *r* = .56 at the item level.

Examination of correlational analyses between the DAPTQ–French version and measurements of perceived stress, trait anxiety, and creativity further support the DAPTQ as a valid and reliable instrument to measure adaptive traits associated to psychopathy. Similar to the results obtained during the DAPTQ development, the DAPTQ–French version correlated strongly negatively with both the PSS and the STAI-Y2 [34]. At the subscales level, the DAPTQ’s Composure and Management subscales displayed the strongest correlation to the PSS, and the Leadership, Composure, Focus, and Management subscale the strongest correlation to the STAI-Y2. These results corroborate the findings obtained during the development of the DAPTQ.

Comparison of the DAPTQ with a range of measures not previously studied in conjunction with the DAPTQ provided additional information regarding the psychometric information of the DAPTQ. First, the DAPTQ was strongly negatively associated with personal distress. The IRI-PD scale has been associated in previous studies to the Boldness component of the TriPM (*r* = -.471) and the PPI-I (*r* = -.52) [54,55]. Second, the PANAS-Positive has been associated positively with PPI-I (*r* = .40), while the PANAS-Negative has been associated negatively with PPI-I (*r* = -.30) [56]. Third, the SWLS has been associated with the TriPM-Boldness (*r* = .35) [57]. Fourth, the SSES has been associated with PPI-I (*r* = .50) [26]. Lastly, while the SCAB has never been used in research related to psychopathy, multiple studies have found weak associations between creativity and psychopathic traits [58–61]. This would suggest that higher scores on the DAPTQ will result in increased enjoyment of working on creative projects, higher creative cognitive abilities, such as divergent thinking and problem solving, increased excitement seeking, and increased flexibility and openness to others’ ideas [62].

There are multiple limitations to the present study. First, recruitment was exclusively centered on individuals residing in France or Canada. Although French is one of the official languages in both of these countries, validation of the DAPTQ–French version in other francophone countries, such as Belgium, Monaco, Côte d’Ivoire, Haiti, and French Polynesia, may provide additional results regarding the cross-cultural stability of the DAPTQ. Second, while French is the only official language in France, Canada’s official languages are French and English. This may influence the results, as the mother tongue of the participants in Canada is evenly distributed between English and French, while the mother tongue of French participants was predominantly French. Third, although self-reported questionnaires are commonly used in the field of personality, experimental evidences are necessary to further support the translated version of the DAPTQ as a reliable instrument to assess adaptive traits associated with psychopathic traits. Fourth, the gender of participants was not evenly distributed in Study 2. During the development of the DAPTQ, a gender difference was noted on the DAPTQ total score, where males displayed higher scores than females [34]. The higher proportion of males in Study 2 may explain why the mean score on the DAPTQ total is higher than the total score in Study 1. Fourth, both studies used a very limited sample size, and results should be replicated in larger samples. Fifth, the current studies did not include instruments assessing social desirability and honesty of the answers. Face to face interviews could allow researchers to identify signs of dishonesty. Sixth, no personality variables that could affect the response style of participants, such as narcissism, have been taken into account. Screening participants with extensive personality questionnaires could provide useful information regarding the desired self-image of participants and its effect on responses. Seventh, the present study did not perform a measurement of invariance of the DAPTQ–French version in both populations due to the small sample size. A future study should examine the results from a confirmatory factor analysis performed in both Canadian and French samples in order to ensure the validity of the current results. Lastly, another limitation focuses on the recruitment method, which was centered on advertising on social media and various websites dedicated to psychological research. Although these recruitment techniques enable to get a wide sample from across both countries, a more specific sample, such as university students from a single university, might provide additional results. Despite these limitations, the present findings are promising as they provide further evidence of the cross-cultural equivalence of the DAPTQ to measure adaptive traits related to psychopathic traits in various populations.
